# Medical Military Service Record

**Published:** 1918-07

**Authors:** 


### Medical Military Service Record.

The Jour. of the A. M. A. estimates the total number of physicians in the country at 144,116, serving a population of 106,543,317. For reason previously stated, we believe that the census of physicians includes a great many who have not been in practice but in some other vocation for some years not to mention those who are more less retired. The estimate of population, based on previous rate of increase is probably also somewhat exaggerated as our increase of about 20% per decade has been due to immigration about as much as to natural increase. However, both figures are substantially correct and indicate one physician to 739 people and to 21.4 square miles of territory—which can be considerably increased without leaving any to suffer from lack of professional care. 4,864 of the physicians are women, not directly available for military service according to precedent though some are already in the military service and the majority have expressed a willingness to serve and have already engaged in activities quite as useful in an indirect way. 107,255 physicians are under 55 and 75,498 under 45. 81,239 are members of county societies. 19,692 are already commissioned—13.7%, the proportions varying from 7.8% for Arkansas to 23% for Arizona, although the former has slightly less than the average clientele and area per physician and the latter and considerably more than the average clientele (797) and area (294.2 square miles).

The figures for N. Y. State and the western counties are as follows:

### NEW YORK

| County | Area Square Miles | Sq. Miles per Physician | Population Est. 1917 | Population per Phys. | Total No. Physicians | Total Women Phys. | Physicians Under 45 | Physicians under 55 | Members of Co. Society | Commis'd in M.R.C., etc. |
|---|---|---|---|---|---|---|---|---|---|---|
| Allegheny ....... | 1,047 | 17.4 | 41,412 | 690 | 60 | 1 | 23 | 29 | 42 | 10 |
| 2Broome ......... | 705 | 4.6 | 95,450 | 523 | 153 | 8 | 62 | 108 | 92 | 19 |
| Cattaraugus ..... | 1,343 | 14.4 | 75,535 | 812 | 93 | 3 | 42 | 59 | 54 | 18 |
| 3Cayuga .......... | 703 | 7.1 | 70,261 | 706 | 99 | 8 | 27 | 64 | 65 | 9 |
| 4Chautauqua ..... | 1,069 | 7.1 | 121,570 | 805 | 151 | 2 | 57 | 98 | 102 | 22 |
| 5Chemung ........ | 407 | 4.4 | 60,786 | 660 | 92 | 7 | 41 | 61 | 56 | 13 |
| Chenango ....... | 894 | 14.9 | 37,083 | 618 | 60 | 3 | 24 | 32 | 38 | 9 |
| Cortland ......... | 503 | 11.2 | 30,469 | 677 | 45 | .. | 12 | 23 | 37 | 3 |
| Delaware ........ | 1,449 | 26.3 | 46,166 | 839 | 55 | .. | 15 | 36 | 26 | 3 |
| 7Erie .............. | 1,034 | 1.1 | 598,549 | 646 | 926 | 41 | 471 | 682 | 651 | 135 |
| Genesee ......... | 496 | 10.3 | 41,963 | 874 | 48 | 3 | 22 | 31 | 37 | 6 |
| Livingston ....... | 631 | 8.9 | 38,752 | 545 | 81 | 6 | 28 | 32 | 50 | 9 |
| 10Monroe .......... | 663 | 1.3 | 339,157 | 654 | 518 | 30 | 270 | 382 | 351 | 83 |
| 13Niagara ......... | 522 | 4.2 | 115,263 | 929 | 124 | .. | 60 | 94 | 73 | 19 |
| 14Oneida ........... | 1,250 | 5.0 | 178,860 | 715 | 250 | 10 | 127 | 184 | 180 | 32 |
| 15Onondaga ....... | 781 | 1.9 | 228,307 | 546 | 418 | 18 | 176 | 301 | 228 | 41 |
| Ontario .......... | 649 | 6.9 | 55,580 | 591 | 94 | 3 | 34 | 60 | 73 | 1 |
| Schuyler ........ | 336 | 13.4 | 14,004 | 560 | 20 | .. | 10 | 14 | 18 | 3 |
| Seneca .......... | 336 | 7.1 | 26,972 | 573 | 47 | 2 | 19 | 31 | 28 | 6 |
| Steuben ......... | 1,401 | 11.5 | 83,755 | 686 | 122 | **5** | **48** | **75** | **82** | **13** |
| Tioga ............ | 520 | 11.8 | 25,624 | 582 | 44 | 1 | 13 | 27 | 23 | 7 |
| Tompkins ....... | 476 | 5.5 | 37,708 | 438 | 86 | 8 | 31 | 54 | 63 | 14 |
| Wayne .......... | 599 | 9.1 | 54,816 | 830 | 66 | .. | 20 | 40 | 38 | 9 |
| Wyoming ........ | 601 | 11.1 | 33,495 | 620 | 54 | 3 | 15 | 31 | 37 | 7 |
| Yates ............ | 343 | 11.1 | 18,922 | 610 | 31 | 1 | 10 | 22 | 22 | 4 |

2. Includes Binghamton, population 55,791; physicians 107 (M.R.C. 14).
3. Includes Auburn, population 37,823; physicians 61 (M.R.C. 8).
4. Includes Jamestown, population 40,415; physicians 48 (M.R.C. 7).
5. Includes Elmira, population 41,278; physicians 79 (M.R.C. 12).
7. Includes Buffalo, population 475,781; physicians 857 (M.R.C. 123).
10. Includes Rochester, population 264,714; physicians 461 (M.R.C. 77).
13. Includes Niagara Falls, population 47,058; physicians 49 (M.R.C. 10).
14. Includes Utica, population 89,272; physicians 150 (M.R.C. 14).
15. Includes Syracuse, population 158,514; physicians 336 (M.R.C. 35).

### Allegheny County

ALFRED—Emerson Winfred Ayars. BELMONT—Ralph Elmer Robinson. BOLIVAR—Laurence Hackett. CANASERAGA—Carl Geo. Schwan. CERES—Frederick John Pflisterer. CUBA—Theron Blain Bond. FRIENDSHIP — Harold MacMurray Johnson. RUSHFORD — Hanford Kendall Hardy. WELLSVILLE—Ray M. Eaton; Frederick Eugene McCarty.

### Broome County

BINGHAMTON—Samuel M. Allerton; Carleton T. Bagley; Raymond Gernand Bell; Myer Sol. Bloom; John D. Bowen; John Groat Corson; Don M. Hooks; Harry Isaac Johnston; Ulysses Silver Kann; Sylcanus James Nunn; Frank W. Sears; Charles Anthony Squires; Daniel C. O'Neil; Lester E. Sanford. ENDICOTT—Dwight Guilford Dudley; James Santee McNett. JOHNSON CITY—John Walter Farrell. PORT DICKINSON—George Jesse Ganow. UNION—Ray Holly Humphrey.

### Cattaraugus County

ALLEGANY—Jay Gould. DELEVAN—Myron Everett Fisher. EAST

RANDOLPH—James Edgar Crossman. GOWANDA—Herman Walter Johnson; Ira Warner Livermore; Frederick Perlee Schenkelberger. OLEAN—James Jay Clark; Clarence Albert Greenleaf; John Alexander Johnson, Jr.; Raymond Bartlette Morris; Benjamin Van Campen; Donald Angus MacDuffie. PERRYSBURG—Arthur Burt Graves. SALAMANCA—John Conrad Hoeffler; Charles Arthur Lawler; James Almon Taggert; George Beckwith Ubel. ALLEGHENY—Eugene Daniel Quinlan.

### Cayuga County

AUBURN—Francis Joseph Bennett; Eugene Napolean Boudreau; John Wordsworth Copeland; Raymond Fleming Johnson; Wm. Henry Kober, Jr.; Rob Roy McCully; Forest Ray Mildren; Maxwell Kemper Willoughby. WEEDSPORT—Clinton Eddy Goodwin.

### Chautauqua County

CASSADAGA—Henry Salem Edmunds. DUNKIRK—George R. Irving; Henry Jos. Meister; Norman Leo Sheene; Walter Hall Vosburg; John Charles Webster. FREDONIA—Roy John Juhre; Harry Elmer Wheelock. FREWSBURG — Francis John McCullo. JAMESTOWN — Frank Perry Goodwin; Floyd Warner Hayes; Walter G. Hayward; Edward Laban Hazeltine; Bergen Fred Illston; Milton John Johnson; William Miller Sill. KENNEDY—George William Batt. RIPLEY—Paul Sterrett Persons. SHERMAN—Guy Granger; Harold Elliott Shaver. WESTFIELD—Gunni Julius Busck; Roswell Fellows Foster.

### Chemung County

ELMIRA—Floyd Pinckney Breese; Charles Hendry Erway; Arthur Clair Glover; Floyd Harding Jones; Louis Eugene McCanna; Edgar Warden Phillips; Stewart Stow Piper; Daniel Edgar Pugh; Arthur C. Smith; Donald Joseph Tillon; Raymond A. Turnbull; Bert Grant Voorhees. VAN ETTEN—Benjamin Franklin Colegrove.

### Chenango County

GUILFORD—Blinn A. Buell. LINCKLAEN—Frederick Dudley Keppel. NORWICH—Edwin Fred Gibson; William Edward Hartigan. OXFORD—Burton Alexander Hall. SHERBURNE—Archibald K. Benedict. SMYRNA—Leroy Dilmore Soper. SOUTH OTSELIC—Jaynes Mott Crumb; Archibald Thomas Perkins.

### Cortland County

CORTLAND—James J. Parsons; Daniel Robert Reilly. VIRGIL—John Harry Evans.

### Delaware County

DELHI—Grover Asa Silliman. DOWNSVILLE—Fred De Grande Wilson. SIDNEY—Ralph Henry Loomis.

### Erie County

BUFFALO—Francis Argus; Lloyd Kenneth Babcock; Antonio L. Barone; Peter Joseph Barone; Samuel Barone; Charles Joseph Barone; George Cosimo Barone; Herbert Henry Bauckus; Lynn Staley Beals; George Adam Becker; Edwin L. Beebee; Lawrence Felvin Belzer; A. L. Benedict; Maynard Gilmore Bensley; Joseph Barton Betts; Plynn Morton Bolton; Joseph Patrick Brennan; Jos. Brumberg; Patrick H. J. Buckley; Boleslaw M. Bukowski; Lorenzo Burrows, Jr.; John Cunning Brady.

O. J. Case; Marshall Clinton; Julius Y. Cohen; Raymond Cleveland Conklin; George Ferdinand Cott; Harold Wm. Culbertson.

David M. Davis; Robert Edward DeCew; Richard Newnham DeNiord; Robert Paul Dobbie; Timothy Francis Donovan; Henry DeWitt Duryea.

Earl Leo Eaton; George J. Eckel; Albert Richard Ellison; John Fitz-Gerald Fairbairn; Lee Francis Masten; Carl Grover Frost; Edward Lycander Frost; Frederick William Filsinger; Urvan Andrew Fischer.

Albert August Gartner; Harvey Russell Gaylord; George John Geisler; Milton Harry Goldberg; John C. Grabau; Irving Franklin Gram; Samuel Grienstein; John George Grotz.

Francis John Haley; George McK. Hall; Barton E. Hauenstein; Raymond Hensel; Wm. Oakley Hill; Robert W. Hinds; Harry Joseph Hammond.

Harold Bartlett Johnson; William Harry Jones; Daniel Jung; Russell Stewart Kidder; Robert King; Chas. Gowen B. Klophel; Clarence Perry Kummer; Frank Kruse; Leon Sebastian Kurek.

Jos. Peter La Duca; Charles W. Lane; Sabrater C. Lojacono; Charles Edward Long; Frank Henry Long; Earl Henry Lormor; William Chisholm Lucas.

Walter Louis Machemer; Wallace F. MacNaughton; Baldwin Mann; Herman Frank May; William Lee McCanty; Arthur Everett McCarthy; Harold James McDonald; Hugh Chauncey McDowell; Descum Clayton McKenney; Michael James McMahon; Roland Otto Meisenbach; Edward Frederick Meister; John Anthony Metzen, Jr.; Rudolph C. Miller; Vincent Chas. Moscato; Jerome Aloysius Murphy; Thomas Henry McKee.

Joseph Aloysius Nowicki; Oscar J. Oberkircher; Geo. Melvin Oppermann; William Ostrow; Frederick Wm. Palmer; Charles C. Panzarella; Harold Alexander Patterson; William Ward Plummer; Frank Nelson Potts; Thomas H. Powick; Leon Hastings Prior.

Nelson Gorham Russell; Leo Marks Sachs; Arthur C. Schaefer; Charles Simon; Herbert Alexander Smith; Edward Archibald Southall; Frederick Edward Sperry; John Gurney Stowe; Fredk. Ettore Strozzi; Jas Cornelius Sullivan.

Earl Wm. Thoma; Carl Tompkins; Haworth R. Traver; Alfred Herbert Vogt; Frank Gebhard Walz; John Henry Watson; Harry Milton Weed; Grover Wm. Wende; Carlton E. Wertz; Hiram Samuel Yellen. COLDEN—Warren Z. Dell. HAMBURG—Amos John Minkel. COLLINS—Herman Lester Raymond. LACKAWANNA—Harry Alexander Scott; Kenneth Allan Smith; Edward A. Twist; Roy B. Woodward. LANCASTER—Clarence Herbert Mackey. SPRINGVILLE—Gregory Everett Stanbro. TONAWANDA—Howard Cousins Fairbanks; Archibald William Thompson. WILLIAMSVILLE—Harry Bestow Huver.

### Genesee County

BATAVIA—Anthony Joseph Greco; Clarence Bentley Gould; Ward Beecher Manchester; Elmer Elwell Owen; Henry Morris Spofford. BYRON—Edgar Bieber.

### Livingston County

AVON—Charles Guy Swan. DANSVILLE—Clark Vernon Fairbanks. GENESEO—Charles I. Newton. LIVONIA—Vernon Leslie Bishop. NUNDA—William Erwin Diefenbach. SONYEA—Elias Cecil Fischbein; Charles Aubrey Joy; James Frederick Munson. YORK—Benj. Harrison Dike.

### Monroe County

FAIRPORT—William Henry Ferrier; George S. Price. HILTON—Walton Hovey. ROCHESTER—Max Alonzo Almy; Frank B. Baldwin; Leon James Barber; Edmund C. Boddy; Anthony Bondi; Charles O. Boswell; John Ruskin Bradley; Raymond John Brown.

Michael Lester Casey; Alfred F. Cassebeer; Milton Chapman; Leighton Randolph Cornman; Joseph Richard Culkin.

Sol Charles Davidson; James Clement Davis; Michael Richard De Vita; John C. Dessloch; Raymond E. Elliott; William V. Ewers.

Ralph Roswell Fitch; James Murray Flynn; John Denison Fowler.

George H. Gage; Frederick Joseph Garlick; George Washington Goler; Philip Gordon; Thomas John Goundry, Corden Thorne Graham.

Edward L. Hanes; Clayton Kendall Haskell; Charles W. Hennington; Joseph P. Henry; Wallace Herriman; Charles L. Hincher; James LeRoy Hondorf; Charles W. Hoyt; Joseph Edward Hurley.

Edwin Stanley Ingersoll; William B. Jones; Albert D. Kaiser; Franklin Austin Knope.

Abraham Lebendig; Macy Levi Lerner; William Alexander Macpherson; Charles Maggio; Henry Elsner Marks; David Ralph Melen; Alvah Strong Miller; Harry Adelbert Miller; Howard D. Mitchell.

Lawrence James Nacey; Francis J. O'Brien; William K. Otis.

Myron Botsford Palmer; Paul Martin Parker; William W. Percy; Norman Jos. Pfaff.

Hiram Randall; Arthur Patterson Reed; Ray D. Richman; Francis John Robinson; Samuel Hendricks Rosenthal.

Jacob Sachs; Harry Allen Sadden; William Waldo Schairer; Benjamin James Slater; Alexander Lorne Smith; Harold Launt St. John; Charles Clyde Sutter; John M. Swan; Frand Sackett Schonover, Jr.

Julius T. Waterman; Edward Tubbs Wentworth; William Richardson Woodbury; Warren Wooden; Harry Wooden; Harry Wronker; Chas. Carmelo Teresi. SULLIVAN—Elsworth Eliot.

WEBSTER—James Byron Foster; William Stanton.

### Niagara County

JOHNSON CREEK—Murray F. Mudge. LOCKPORT—Lemuel Rankins Hurlbut; Henry Hamilton Mayne; Frank Austin Walder; Lyman Hall Wheeler. NEWFANE—Edwin Shoemaker. NIAGARA FALLS—Raymond Samuel Barry; John Lewis Bishop; Charles C. Childs; Edward Eugene Gillick; Howard Joseph Hutter; Frederick N. C. Jerauld; Robert J. Lawler; Ernest Martin Guido-Rieger; John Preston Sharp; Francis Joseph Talbot. NORTH TONAWANDA—Harry Oren Maldiner; Martin Francis Nalon. YOUNGSTOWN—Lewis William Falkner.

### Oneida County

CAMDEN—George Canfield Lyons. CLARK MILLS—Fred Goodwin Jones. CLINTON—Roy Bicknell Dudley; Varney Bernard Hamlin; Erwin G. MacFarland. NEW YORK MILLS—Henry Miller Mitchell. ORISKANY FALLS—Robert Bruce Wilson. ROME—William Lester Grogan; Ray Jay Marshall; Maxwell Comrie Montgomery; William Bradley Reid; Le Clare Stuart; Kent Eugene Williams. UTICA—Charles H. Baldwin; George B. Campbell; Francis Temple Chase; Jason Henry Conger; Frederick Christopher Devendorf; Arthur Rogers Grant; Miles Wendel Johns; John Joseph Leary; Harold Cleveland Lyman; Stephen A. Mahady; Nicholas Alfred Sullo; Robert Francis Zimmerman; John Richard Fischer; Frederick Thomas Owens. VERNON—John Dean Shipman. WATERVILLE—Lewis Furbeck Cole; Edward Gove Randall; Howard David MacFarland.

UTICA—John Richard Fischer; Frederick Thomas Owens.

### Onondaga County

CAMILLUS—George Hamell Shaw. JORDAN—Walter W. Osgood. MANLIUS—R. M. Ballantyne. MOTTVILLE—Milton Edward Gregg. POMPEY—Frederick Austin Hunt. SYRACUSE—Adelbert Cleveland Abbott; William Dewey Alsever; Archer D. Babcock; Clyde O. Barney; Arthur W. Brennan; George Sidney Britten.

Murray A. Cain; Donald Smythe Childs; Clarence E. Coon; John James Corbett.

Raymond John Devine; Brewster Clarke Doust; Henry Burton Doust.

Scott Romain Fisher; Albert Alton Getman; Herbert Gifford.

Lee Arthur Hadley; Herbert Israel Kallet; Jefferson B. Latta; Glendon Richard Lewis; Daniel F. Luby.

Henry Alexander MacGruer; Brooks Walton McCuen; Arthur Dubois Meyers; Bernard Stanislaus Moore; Leonard Stevens Nolan.

Philip Sheridan Potter; Robert Case Scott; Myles Bernard Sharkey; George Kellogg Smith; Samuel Stewart; Earl Vincent Sweet; William E. Truax.

Edward Seguin Van Duyn; Earle Cyrenius Winsor; Edward Judson Wynkoop.

**Ontario County**

CANANDAIGUA—Alfred W. Armstrong; Frederick Coe McClellan. CLIFTON SPRINGS—Samuel Darragh Earhart; Samuel Timothy Nicholson, Jr.; Walter Stevens Thomas; Walter Longfellow Weeden; John Alexander Wentworth. GENEVA—James Stevenson Allen; Isaac Williams Brewer; Thomas W. Maloney; Frank Hassan Snyder; Thomas William Maloney. GORHAM—Lloyd Frederick Allen. SHORTSVILLE—Daniel Alverson Eiseline. STANLEY—Charles W. Selover.

**Schuyler County**

ALPINE—John William Burton. VALOIS—Palmer H. Lyon. WATKINS—Noah Philip Norman.

**Seneca County**

OVID—John Graham Gordon. SENECA FALLS—Robert Knight; Frederick William Lester. WATERLOO—Francis Victor Hoehn; William Wallace Carleton. WILLARD—John Francis McNeill.

**Steuben County**

BATH—Raymond Coleman Hill. CANISTEO—Harold Hubbard Mitchell. CHOCTON—John H. Miller. CORNING—Willis Sylvester Cobb; Ralph Francis Gregorius. HORNELL—Milton Gardner Burch; James Raymond Kelly; Leon M. Kysor; Frederick Carver Robbins; George Ellis Taylor; William Joseph Tracy; Clark Anson Wilcox. JASPER—Leon Mitchell Wilbor.

**Tioga County**

NICHOLS—Leroy Jakway Osborne. OWEGO—Kennedy Furlong Rubert; Sidney Welles Thompson. SPENCER—Frederick Terwilliger. WAVERLY—Paul Eugene Betowski; Arthur John Smith; Frederick Hallet Spencer.

**Tompkins County**

ENFIELD CENTER—David Robb. GROTON—James Howard Van Marter. ITHACA—John Frank W. Allen; Reed Brinsmade Bontecon; Albert Cyrus Durand; Chas. Henry Gallagher; Walter Bonnell Holton; Minor McDaniels; Samuel Archer Munford; Martin Buel Tinker; Isador Mack Unger; Otto Emil Utzinger; Royden Mendeville Vose. LUDLOWVILLE—Ira A. Allen.

**Wayne County**

ALTON—William George Lewis. LYONS—Reuben Spencer Simpson. MARION—Arthur Besemer. NEWARK—Louis Stone Kelley; John Andrew Morrisey; Cyrenius Adelbert Newcomb; James Raymond Sanford. PALMYRA—Lee Rice Pierce. WOLCOTT—Samuel Wilson Houston.

**Wyoming County**

ATTICA—Willard DeForest Preston. CASTILE—Harold Elmer Foster. GAINESVILLE—George Stephen Skiff. PERRY—Thos. M. Calladine, Jr.; Milton Oren Houghton. WARSAW—James Frank Crawford. WYOMING—Arthur Willard Hubbard.

**Yates County**

BRANCHPORT—John Henry Rose. PENN YAN—Edwin Carlton Foster; Herbert Wood Matthews; Bernard Samuel Strait.

The names are given to June 1. Our readers are urged to preserve this list, inform us of any corrections and to preserve the supplementary lists which will be published from time to time.

It will be noted that about a third of all physicians under 55 are between 55 and 45 and this proportion seems to hold good for the men in military service, indicating that the physical condition, desire for service and ability to make the necessary financial sacrifice is equally great at advanced ages.

